# *Mycoplasma genitalium*: An Emerging Sexually Transmitted Infection

**DOI:** 10.1155/2016/7537318

**Published:** 2016-02-29

**Authors:** Jessian L. Munoz, Oluwatosin Jaiyeoba Goje

**Affiliations:** Ob/Gyn and Women's Health Institute, Cleveland Clinic, 9500 Euclid Avenue A81, Cleveland, OH, USA

## Abstract

*Mycoplasma genitalium* has been recognized as a cause of male urethritis, and there is now evidence suggesting that it causes cervicitis and pelvic inflammatory disease in women.* M. genitalium* is a slow growing organism, and, with the advent of nucleic acid amplification test (NAAT), more studies are being performed, and knowledge about the pathogenicity of this organism elucidated. With NAAT detection, treatment modalities have been studied, and the next challenge is to determine the most effective antimicrobial therapy. Doxycycline, the first-line antibiotic for urethritis, is largely ineffective in the treatment of* M. genitalium* and furthermore, resistance to macrolide has also emerged. The most effective drug is Moxifloxacin although there are emerging reports of resistance to it in various parts of the world. This paper not only highlights the current research and knowledge, but also reviews the diversity of health implications on the health of men and women infected with* M. genitalium*. Alternate antibiotics and the impact of* M. genitalium* on infertility are areas that require more studies as we continue to research into this microorganism.

## 1. Introduction

*Mycoplasma genitalium* is a slow growing organism, and the advent of nucleic acid amplification test (NAAT) has shed more light on this emerging sexually transmitted infection (STI). There has been an increase in interest, research, and knowledge about* M. genitalium* in recent years. The most recent Centers for Disease Control and Prevention (CDC) sexually transmitted diseases treatment guidelines, published in 2015, discuss* M. genitalium* under emerging issues [1]. It is therefore imperative that both scientist and clinicians understand the organism, pathogenicity, and sequelae.

*M. genitalium* infection occurs globally, having been found in every country where it has been sought. The prevalence of* M. genitalium* varies within countries and communities. A large study in the Netherlands documented a 4.5% prevalence in their community, second only to* Chlamydia trachomatis* (8.3%), and greater than* Neisseria gonorrhoeae* (1.3%) and* Trichomonas vaginalis* (1.4%) [2]. In Sweden, 6.3% of patients at a sexually transmitted diseases (STD) clinic were noted to be infected with* M. genitalium*, with 45% of these patients having partners who also harbored the pathogen [3]. In England, the prevalence of* M. genitalium* was considerably lower (1.2%) [4]. A study from the United States showed a prevalence of 0.4% in young adults [5]. In that study,* M genitalium* was more prevalent than* N. gonorrhoeae* but less prevalent than* C. trachomatis*, and it was strongly associated with sexual activity.

*M. genitalium* was detected in 5.8% of human immunodeficiency virus (HIV) positive men by PCR analysis in Brazil [6]. Mavedzenge et al. documented that it may be an independent risk factor for the acquisition of HIV-1 in Zimbabwe and Ugandan women [7]. In addition,* M. genitalium* was also associated as a cofactor for HIV infection in a case-control study of Ugandan women [8].

There exist many barriers and gaps to further understand* M. genitalium* infection and its impact on human health. Currently, there is no uniform method of detection of* M. genitalium*, which makes a collective comparison difficult. In addition, the precise role of* M. genitalium* in disease such as pelvic inflammatory disease, infertility, and pregnancy is not currently known, and more studies are needed.* M. genitalium* treatment has also shown to be difficult given mechanisms of resistance and variation in clinical management. These are gaps we highlight in this review and compare the data available to assess the importance of* M. genitalium* to disease processes and treatment. To analyze the literature with respect to* M. genitalium* in human pathology, we reviewed articles available on PubMed in the English language from 1981 to 2015, which explored* M. genitalium* as a human pathogen.

## 2. Diagnostic Consideration

A significant step in* M. genitalium* research would be uniform detection of acute infection and prior exposure. Isolation and culturing of* M. genitalium* is slow, time consuming, and not feasible when there is a need to institute immediate antimicrobial therapy. Therefore, NAAT is the preferred diagnostic method where feasible. Although research companies have quantitative PCR detection kits in the market, the United States Food and Drug Administration (FDA) has not approved any of these methods for the clinical screening or detection of* M. genitalium*. Vandepitte and colleagues [8] compared two commercially available kits (TIB MOLBIOL LightMix kit and the Diagenode* M. genitalium* real-time PCR kit) as well as an in-house PCR method using the Roche Diagnostics cobas z 480 analyzer [9]. TIB MOLBIOL LightMix kit targeted the* mg219* gene, Diagenode* M. genitalium* real-time PCR kit targeted the* gap* gene, and the in-house kit targeted the* MgPa1* adhesion protein gene. The commercial kits had a sensitivity of 92.6% and 87%, respectively, and a specificity of 100% which was concordant with the in-house kit that was >95%.

In an effort to establish a simpler and streamlined protocol for* M. genitalium* detection, Takanashi and colleagues developed a PCR test using InvaderPlus® technology, carrying out both the endonuclease and PCR in the same simple step [10]. This approach would require less genetic material and would be of less labor and would be time consuming. The approach was tested with first-void urine samples and the PCR target was the 16S rRNA gene of* M. genitalium*. The InvaderPlus assay was comparable to typical hybridization microtiter PCR, able to detect as few as 10 DNA copies per reaction. A 99.3% concordance between the two assays was noted (137/138). While this assay was not tested with urethral, pharyngeal, vaginal, or anorectal swabs for clinical determination, the data was found to be promising.

Another opportunity for biotechnology in* M. genitalium* pathology is establishing genetic markers of resistance to first-line therapy. Currently,* M. genitalium* antibiotic therapy reflects* C. trachomatis* therapy (Doxycycline and Azithromycin). Resistance to azithromycin through mutations in ribosomal genes has been reported [11]. In France, a combination of PCR and FRET analysis revealed 14.2% of samples contained antibiotic-resistance associated mutations of the* 23 rRNA* gene, but no correlation with treatment was established [12]. By screening the* 23 rRNA* gene in Australia, it was noted that 20% of pretreatment and 100% of treatment failure samples contained mutations of this gene which may confer resistance to macrolides [13]. Therefore,* M. genitalium* may have intrinsic as well as induced mechanisms of resistance to antibiotic therapy.

## 3. Clinical Considerations

### 3.1. *M. genitalium* in Men

Although* Neisseria gonorrhoeae* and* Chlamydia trachomatis* are well known causes of male urethritis,* M. genitalium* has arisen as another cause. In 1981,* M. genitalium* was isolated for the first time from the samples of 2 men with nongonococcal urethritis (NGU) [14], and since then, data supporting the role of* M. genitalium* as a cause of male urethritis has increased over the years. Studies showed that* M. genitalium* infection had a 6.5-fold increased risk of urethritis (22% versus 4% of controls, 95% CI 2.1–19.5), after controlling for* N. gonorrhoeae* and* C. trachomatis* [15].* M. genitalium* has also been implicated in balanitis and posthitis. In a study of 114 men with nongonococcal urethritis,* M. genitalium* significantly correlated with the development of balanitis and posthitis [16]. Conversely, sperm concentration was negatively correlated with* M. genitalium* infection [17].

There is conflicting data on* M. genitalium* and circumcision. A study on male circumcision in Kenya revealed* M. genitalium* was detected in 13.4% of uncircumcised men compared with 8.2% of circumcised men (*p* = 0.06). Adjusted odds ratio showed a 50% reduction in* M. genitalium* infection when men were circumcised [18]. On the other hand, a study from England, did not detect a relationship between* M. genitalium* and circumcision, although it was underpowered [19].

Trends in oral and anal sex have increased over the past decades; anal intercourse has doubled over a 10-year period [20]. A study of 1778 men screened by urine and anorectal swabs revealed 91 (5.1%) were positive for* M. genitalium*. Of note, 71.4% of* M. genitalium* positive patients' in the study had positive anorectal swab. [21]. Another study using anal swabs reported 4.2%* M. genitalium* positive screen among HIV positive men who have sex with men [22].

In addition, 24.3% of* M. genitalium* infected women were noted to have positive anorectal swabs [23].* M. genitalium* has been found in the anorectal region but its pathogenicity in causing clinical proctitis has not been elucidated and more research is required.

### 3.2. *M. genitalium* in Women

The incidence of* M. genitalium* and* C. trachomatis* is relatively similar in high-risk women. In a cross sectional study at an STD clinic,* M. genitalium* and* C. trachomatis* were detected in 6% and 10% of women, respectively [24]. These women presented with cervicitis, and, in addition,* M. genitalium* was detected in 59% of their male partners. The organism has also been implicated in Pelvic Inflammatory Disease (PID);* M. genitalium* was detected in 13% of PID patients and 0% of their controls [25]. It is important to note that the CDC recommended antibiotic regimens for PID, not effective against* M. genitalium* [26].

Although* N. gonorrhoeae* and* C. trachomatis* are known causes of mucopurulent cervicitis, a significant proportion of cervicitis are of unknown etiology [27]. Manhart and colleagues explored* M. genitalium* as a cause of mucopurulent cervicitis [28] and reported* M. genitalium* in 7.0% of 719 women. In addition, risk factors for* M. genitalium* cervicitis were determined, and they include younger age, multiple sexual partners, and a history of miscarriage, smoking, or douching. Thus it was concluded that* M. genitalium* might be a cause of mucopurulent cervicitis.

Furthermore,* M. genitalium* was present in 16% of endometrial biopsies performed in 58 women with histologically confirmed acute endometritis [28]. Known potential complications of endometritis include infertility, pelvic peritonitis, abscess formation, and sepsis.

Given that* M. genitalium* is associated with endometritis, cervicitis, and PID,* M. genitalium* may have a significant effect on reproductive health and pregnancy outcomes. Unlike* C. trachomatis*, some studies using PCR have shown a correlation with ectopic pregnancy but others using serological markers have not shown this relationship [29, 30]. In addition,* M. genitalium* infection does not correlate with early miscarriage [31] but has been associated with increased risk of preterm delivery.* M. genitalium* was associated with preterm labor in a study involving 667 Peruvian women who underwent preterm labor [32].* M. genitalium* was not associated with any other risk factors of preterm labor [31]. Another study of 134 women from the United States revealed a preterm labor rate of 28% and* M. genitalium* was detected in 20.2% of these patients [33]. Taken together, these studies show a relationship between* M. genitalium* infection and the female physiology, pregnancy, and pathophysiology.

### 3.3. *M. genitalium* and Infertility

Approximately 9% of conceiving couples may experience infertility [34], while patency of fallopian tubes, disorders of ovulation, and sperm function are common, bacterial infections also play a role in infertility [35, 36]. In vitro studies confirmed* M. genitalium* can bind to fallopian tube epithelium [37]. An Iranian study using PCR (*16S rRNA*) to detect* M. genitalium* found the pathogen in 2.8% cervical swabs of infertile women [38]. Interestingly, in another study,* M. genitalium* was detected in samples collected from both cervical swabs as well as abdominal laparoscopic washings of women with infertility [39]. The studies suggest* M. genitalium* might play a role in female infertility. Conversely, the possible role of* M. genitalium* in male fertility needs further research. A large meta-analysis which included the data from studies including 307 men with infertility, suggested a minimal role for* M. genitalium* in male infertility [40]. Thus, more research is required to establish a clear role between* M. genitalium* infection and infertility.

## 4. Antibiotic Therapy

The current treatment guidelines for NGU are oral Doxycycline 100 mg twice a day for 1 week or single dose oral Azithromycin 1 g. NGU may also be caused by* M. genitalium* and Doxycycline is ineffective in eradicating* M. genitalium* infection, with efficiency ranging from 17 to 90%, irrespective of macrolide resistance [41]. In addition, with increasing resistance, azithromycin has become progressively less effective [42]. Resistance to macrolides has been reported as high as 30%–40% in certain populations [43]. Furthermore, to test efficacy, the establishment of a time period for test of cure (TOC) is required; the exact time to eradication of* M. genitalium* after treatment is variable. One study showed 96% eradication within 8 days after Azithromycin therapy, as measured by PCR [44]. Interestingly enough, these authors also noted those patients with resistant strains were detected after a period of negative tests, thus suggesting test of cure should be held until 3-4 weeks after treatment. When treated with Moxifloxacin, PCR testing was negative within a week. In this study by Falk and colleagues, doxycycline treatment did not eradicate* M. genitalium* in 6 of 8 patients in this study.

Extended azithromycin therapy has been suggested as an alternative method for antibiotic therapy and to reduce the development of resistant strains [45]. To explore the efficacy of this extended treatment 54 females and 31 males were treated with oral Azithromycin 500 mg on day 1 and 250 mg on the following 4 days; test of cure (TOC) analyses were performed 6 weeks later; 25% of patients still tested positive for* M. genitalium* at TOC [46]. The greater risk of treatment failure is the development of macrolide resistant strains in the community.

Attempts to explore the use of other antibiotics for* M. genitalium* eradication have been made difficult because* M. genitalium* lacks a cell wall thus; it is inherently resistant to antibiotics targeting cell wall synthesis such as beta-lactams and penicillins. Oral Moxifloxacin (dose of 400 mg daily for 7–10 days), a member of the fluoroquinolone family, has shown significant efficiency in* M. genitalium* eradication of macrolide resistant strains. The drawbacks of Moxifloxacin usage are the broad spectrum, side effects, the cost in certain countries, and contraindication in pregnancy. In addition, mutations associated with fluoroquinolone resistance (*parC* or* gyrA*) genes have been reported in 15% of patient samples prior to treatment [47]. Overall, in the management of* M. genitalium* urethritis and cervicitis, doxycycline has poor response compared to Azithromycin. Moxifloxacin has been found to be more effective in patients who have failed previous therapy.* M. genitalium* should be suspected in persistent or recurrent urethritis, cervicitis, and PID. Patients who fail the CDC recommended therapy for PID treatment should be treated using Moxifloxacin for 14 days, and where available, clinicians may test women who have failed recommended PID treatment for* M. genitalium* and treat them accordingly if positive [1].

## 5. Conclusions

Studies demonstrate that* M. genitalium* is an emerging sexually transmitted infection. Additional research is needed regarding pathogenicity and treatment, and there is a need for a standardized NAAT for clinical detection and resistance. Treatment for* M. genitalium* infection should be considered when patients fail first line treatment for urethritis, cervicitis, and PID. In the United States, most treatments will occur in the context of syndromic management of persistent or recurrent urethritis, cervicitis, and PID until we have an FDA approved diagnostic method. If* M. genitalium* diagnosis is performed in communities where NAAT testing is available, Azithromycin extended regimen (500 mg day 1, 250 mg days 2–5) may be considered as first line treatment, but Moxifloxacin is the appropriate drug in patients with previous treatment failure. In addition, HIV positive patients should be treated with the same antimicrobial therapy regimen as HIV negative patients. Sex partners should be treated according to guidelines for patients with NGU, cervicitis, and PID [1].
